# Supplementary material for: Deep learning-based approach for high spatial resolution fibre shape sensing
Source: Commun Eng. 2024 Jan 27;3:19. doi: 10.1038/s44172-024-00166-8 (PMC10955859; doi:10.1038/s44172-024-00166-8)
Supplement: Supplementary file 2 — Description of Additional Supplementary Files [file 44172_2024_166_MOESM2_ESM.pdf]

Description of Additional Supplementary Files

File name: Supplementary Movie 1

Description: This video visualizes examples of the sensor's estimated shape using the MFD and the DL methods. Examples are chosen from the **Test_1** dataset.

File name: Supplementary Movie 2

Description: This video visualizes examples of the sensor's estimated shape using the MFD and the DL methods. Examples are chosen from the **Test_2** dataset.

File name: Supplementary Movie 3

Description: This video visualizes examples of the sensor's estimated shape using the MFD and the DL methods. Examples are from the **Test_3** dataset.
